# Chloroquine-Enhanced Efficacy of Cisplatin in the Treatment of Hypopharyngeal Carcinoma in Xenograft Mice

**DOI:** 10.1371/journal.pone.0126147

**Published:** 2015-04-29

**Authors:** Xing-guo Zhao, Rui-jie Sun, Xiao-yan Yang, Da-yu Liu, Da-peng Lei, Tong Jin, Xin-liang Pan

**Affiliations:** 1 Department of Otolaryngology, Qilu Hospital, Shandong University, Jinan, Shandong Province, China; 2 Key Laboratory of Cardiovascular Remodeling and Function Research, Qilu Hospital, Shandong University, Jinan, Shandong Province, China; National Cheng Kung University, TAIWAN

## Abstract

Hypopharyngeal squamous cell carcinoma (HSCC) has the worst prognosis among head and neck cancers. Cisplatin (DDP)-based chemotherapy is an important part of multimodal treatments. However, resistance to DDP severely impairs the effectiveness of chemotherapy for HSCC. Chloroquine (CQ) has been reported to enhance the effectiveness of chemotherapy and radiotherapy in liver, pancreas, breast, prostate and colon tumors, but it is unclear whether CQ could increase the efficacy of DDP for treating HSCC. We inoculated BALB/c nude mice with a subcutaneous injection of human hypopharyngeal FaDu cells to generate our animal model. Mice were randomly divided into 4 groups and treated with vehicle control, CQ (60 mg/kg/day), DDP (5 mg/kg/6 days), or a combination of DDP and CQ. Tumor growth and survival of the mice were monitored. We found that CQ inhibited autophagy and increased DDP-induced apoptosis in the xenograft mouse model. CQ enhanced the efficacy of DDP, resulting in decreased tumor growth and prolonged survival of the mice. To test whether blocking autophagy enhanced the efficacy of DDP, FaDu cells were infected with lentiviral shRNA to Beclin-1 and inoculated into the flanks of nude mice. Inhibition of autophagy markedly enhanced the DDP-induced antitumor effect. Our study suggests that the addition of CQ to DDP-based chemotherapy could be a potential therapeutic strategy for treating HSCC, and the inhibition of autophagy may contribute to chemotherapy sensitization in HSCC.

## Introduction

Hypopharyngeal squamous cell carcinoma (HSCC) accounts for approximately 3 to 5% of all head and neck cancers. The prognosis of HSCC is very poor, and the 5-year overall survival rate is approximately 15 to 45% [1,2,3]. Cisplatin (DDP)-based chemotherapy is an important part of the multimodality treatment for head and neck cancers [4,5]. However, intrinsic and acquired resistance to DDP is common in HSCC treatment, and the effectiveness of chemotherapy is often severely compromised [6]. Over the decades, it has remained difficult to effectively overcome DDP resistance in chemotherapy for head and neck cancers.

Chloroquine (CQ) is widely used as an anti-malarial and anti-rheumatoid drug [7]. Recently, CQ has been reported to enhance the efficacy of drugs and radiation in antitumor studies, including studies on prostate cancer [8], malignant peripheral nerve sheath tumor [9], hepatocellular cancer [10,11,12], colon cancer [13], breast cancer [14] and esophageal cancer [15]. CQ significantly suppressed the growth of pancreatic cancer *in vitro* and *in vivo* as a mono-drug therapy [16]. In contrast, CQ did not sensitize 4T1 tumors [17] or small cell lung cancers [18]. The CQ-induced enhancement of the antitumor effect seems to depend on the tumor type and context. It is unclear whether CQ could enhance the efficacy of DDP in treating HSCC.

The CQ-mediated enhancement of antitumor efficacy has mainly been attributed to its autophagy inhibition mechanism, as reported in the aforementioned literature. Autophagy is a cellular homeostatic process in which cytoplasmic components are sequestered by double-membrane structures and then transported to lysosomes for degradation and recycling [19]. During the process of autophagy, the formation of an autophagosome (a double-membrane cytosolic vacuole that characterizes autophagy) is associated with conversion of the cytosolic-type microtubule-associated protein light chain 3 (LC3) to the membrane-bound type LC3-II. The level of LC3-II is correlated with the extent of autophagosome formation [20]. The adaptor protein p62 sequestosome 1 (p62) can bind directly to LC3 to facilitate degradation of ubiquitinated protein aggregates by autophagy [21]. The accumulation of p62 is associated with blocked autophagy [22]. CQ inhibits autophagy because it can affect lysosome acidification [23]. In studies of autophagy, CQ and its analogs are often used to inhibit the degradation of LC3-II and p62 proteins to measure the autophagic flux. These are the only autophagy inhibitors that can be used clinically.

The role of autophagy in cancer is complex and paradoxical. Autophagy defects can lead to increased tumorigenesis [24,25], whereas autophagy itself can promote the survival of cancer cells under stressed conditions and even facilitate tumor metastasis [26,27]. Previously, we reported that the levels of Beclin-1 (a key autophagy regulator) and LC3 were downregulated in human HSCC [28], indicating an altered autophagy level in hypopharyngeal cancer cells. In the present study, we combined DDP and CQ, an autophagy inhibitor, as an anticancer therapy in a xenograft mouse model with the goal of improving the treatment of human HSCC.

## Materials and Methods

### Ethics Statement

All animal studies complied with the Management Rules of the Chinese Ministry of Health and were approved by the Ethical Committee of Qilu Hospital at Shandong University.

### Cell Culture

The human hypopharyngeal FaDu cell line was obtained from the American Type Culture Collection. Cells were cultured in RPMI 1640 supplemented with 10% fetal bovine serum and 1% penicillin-streptomycin (Invitrogen, Carlsbad, CA, USA) at 37°C and humidified 5% CO_2_.

### Reagents and antibodies

Cisplatin and chloroquine were obtained from Sigma (St. Louis, MO, USA). The antibodies used in the experiments included: rabbit antibodies against LC3, Beclin-1, Bax and β-actin (Cell Signaling Technology, Beverly, MA, USA); rabbit anti-p62 (Proteintech, Chicago, USA); and rabbit anti-Bcl-2 (Abcam, Cambridge, UK).

### Animal Husbandry and Mouse Xenograft

Nude BALB/c mice (5–6 weeks old; male; Beijing Laboratory Animal Research Center, Beijing, China) were maintained in groups of four per cage with food and water available ad libitum in a pathogen-free environment with a 12 h light and 12 h dark cycle. The animals were acclimated 2 days before use and maintained throughout under standard conditions: 22°C ambient temperature and 50% relative humidity.

Human hypopharyngeal FaDu cells were used as a xenograft model in male BALB/c nude mice (5 to 6 weeks old). A suspension of 3 × 10^6^ cells in 100 μL volume was inoculated subcutaneously into the right flank of mice. The tumor sizes averaged approximately 5 × 5 mm after 7 days; then, the mice were divided into 4 groups that were matched for tumor volume and treatment was initiated (7 mice per treatment group). Treatment groups consisted of vehicle control (normal saline); CQ; DDP and DDP + CQ. CQ was administered at doses of 60 mg/kg for 18 consecutive days. DDP was administered at doses of 5 mg/kg every 6 days. 3 injections of DDP were given in total. In the DDP+CQ group, DDP was given 20 min after CQ administration. All the drugs were given by intraperitoneal injection. After 18 days of treatment, mice were sacrificed and tumor tissues were harvested for study (S1 Fig). A caliper was used to measure the tumors every 3 days. The tumor volume was calculated using the following formula: volume = (length × width^2^)/2. The body weight of mice was measured every 3 days to evaluate the systemic toxicity of the drugs. For survival analysis, mice in the CQ group and DDP+CQ group continued to receive administration of CQ at doses of 60mg/kg until they meet the death criteria. Mice were euthanized and considered dead when 1) a tumor exceeded 2 cm in the maximal dimension, 2) a tumor began to cause skin ulceration and 3) a tumor caused the mouse to become moribund [16]. The conditions of the mice were closely monitored (at least 4 times per day). Mice were sacrificed by anesthetizing with intraperitoneal injection of 0.8% pentobarbital sodium (60 mg/kg), followed by cervical dislocation. All efforts were made to reduce pain experienced by the mice. For Beclin-1 shRNA studies, FaDu cells were infected with lentiviral shRNA to Beclin-1 or a scrambled control (GenePharma, Shanghai, China) and subjected to a short puromycin selection; then, 3 million tumor cells were injected into the flanks of nude mice. 7 days after the inoculation, mice were divided into 4 groups (n = 7) matched for the tumor volume: control shRNA group, Beclin-1 shRNA group, DDP+control shRNA group, DDP+Beclin-1 shRNA group. DDP was given at doses of 5 mg/kg every 6 days and 3 injections were used in total. After treatment for 18 days, tumor tissues were collected for analysis. Measurements were performed as described above. All animal experiments were repeated once.

### Western blot

Tumor tissues were lysed in RIPA buffer (50 mM Tris, 150 mM NaCl, 1% Triton X-100, 1% sodium deoxycholate, 0.1% SDS, 1 mM PMSF, pH 7.4) and the supernatant was retrieved. The protein concentrations were determined with a BCA protein assay kit (Beyotime, Beijing). Total proteins were separated by a 10 or 12% SDS-PAGE gel and then transferred onto a PVDF membrane. Membranes were blocked with 5% non-fat dry milk and incubated overnight with various primary antibodies at 4°C. The blots were incubated in appropriate horseradish peroxidase-conjugated secondary antibodies for 1 hour at room temperature. The antigen-antibody complexes were detected by ECL Prime Western blotting detection reagent from GE Healthcare (Pittsburgh, PA, USA). β-Actin was used as a control to monitor the variability in protein loading. Image J 1.48 (National Institutes of Health, USA) was used to quantify the blots.

### Immunohistochemistry (IHC)

Formalin-fixed paraffin-embedded mouse tumor samples were sectioned to a 5-μm thickness and mounted on microscope slides. Antigen retrieval was performed by microwave heating for 15 minutes in 0.01 M sodium citrate buffer (pH 6.0). Slides were washed, treated with 3% H_2_O_2_ and blocked with 5% goat serum for 30 min at 37°C. Tissue sections were incubated in primary antibodies at 4°C overnight; horseradish peroxidase-conjugated secondary antibodies were added and maintained at 37°C for 30 min. A DAB horseradish peroxidase color development kit (ZSGB-Bio, Beijing) was used for positive staining. Tumor sections were observed under a Leica light microscope (DM2500, Germany). Positive areas were quantified by Image-Pro Plus version 6.0 (Media Cybernetics, USA).

### TdT-mediated dUTP nick end labeling (TUNEL)

TdT-mediated dUTP nick end labeling (TUNEL) was performed with an ApopTag Plus Peroxidase In Situ Apoptosis Detection Kit (Merck Millipore, Darmstadt, Germany) according to the manufacturer’s instructions. The number of TUNEL-positive cells was counted and averaged across 10 random fields from 7 mice per group.

### Statistical analysis

Data were analyzed with Prism 5.0 (GraphPad Software Inc., USA). Comparisons were made with one-way analysis of variance or the two-tailed t-test. Kaplan-Meier curves for the survival of mice were analyzed with the log-rank test. Results were presented as the mean ± SEM. A *P* value<0.05 was considered statistically significant.

## Results

### CQ increased the efficacy of DDP, decreasing tumor growth and prolonging the survival of mice

**Fig 1:**
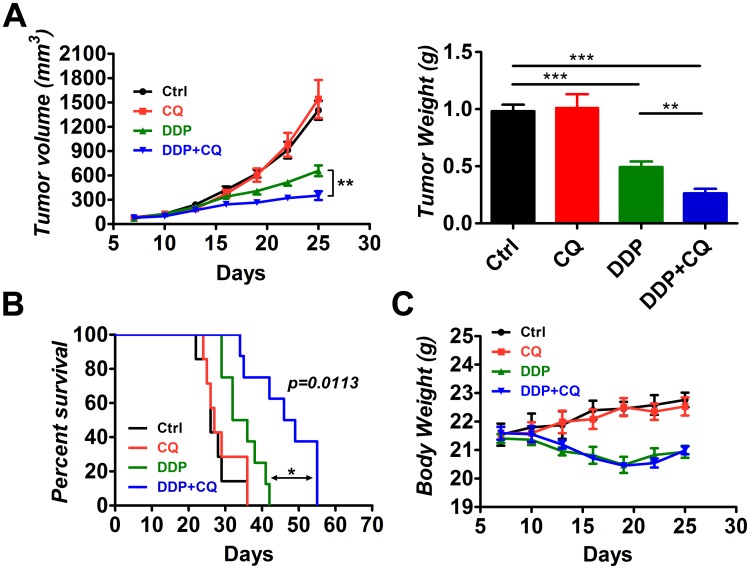
Chloroquine (CQ) enhances the efficacy of cisplatin (DDP) in xenograft tumors. Tumor-bearing mice (7 animals per group) were treated by intraperitoneal injection as follows: vehicle control, CQ (60 mg/kg/day), DDP (5 mg/kg/6 days) and a combination of DDP and CQ for 18 days. (A) The tumor volume and tumor weight for each group. (B) Survival analysis of the treated mice in each group (n = 7). DDP+CQ led to a 15.5-day increase in the median survival compared with the mono-DDP treatment. (C) The body weight of mice during treatment. *p<0.05, **p<0.01, ***p<0.001.

To investigate whether CQ could increase the efficacy of DDP in vivo, male BALB/c nude mice (5–6 weeks old) were used as an animal model. The mice were inoculated subcutaneously with 3 millions of human hypopharyngeal FaDu cells. The mice were divided into 4 groups (n = 7) 7 days after inoculation matched for tumor volume. Mice in groups were treated with vehicle control, CQ (60mg/kg/day), DDP (5mg/kg/6days), or a combination of DDP and CQ. Mice were sacrificed after treatment for 18 days and tumor tissues were collected. We monitored the tumor volume and final tumor weight for each group (Fig 1A). The mono-CQ therapy had no impact on the tumor volume or tumor weight compared with the control group. The mono-DDP group had a decreased tumor volume (657.0±66.2 mm^3^) and tumor weight (0.493±0.0496 g) compared with the control group (1405.0±117.2 mm^3^, 0.981±0.0577 g, *p*<0.001 for both). The DDP+CQ treatment demonstrated a further decrease in the tumor volume (350.7±54.0 mm^3^) and tumor weight (0.263±0.0405 g) compared with the mono-DDP therapy (*p*<0.01 for both).

Kaplan-Meier curves were plotted to evaluate the survival of mice in each group (Fig 1B). The mono-CQ therapy had no effect on survival of the mice, and the mono-DDP therapy increased survival of the mice compared with that of control (*p*<0.01). The combination of CQ and DDP significantly increased survival of the mice compared with that of the mono-DDP treatment (*p* = 0.0113 by log-rank test). DDP+CQ led to a robust 15.5-day increase in the median survival compared with that of DDP alone.

The body weight of xenograft mice was also monitored to evaluate the systemic toxicity throughout the treatment period (Fig 1C). CQ did not induce a loss of body weight compared with the control group. DDP caused a significant loss of body weight relative to the control group (*p*<0.001). DDP+CQ produced a notable body weight loss compared with that of the control group (*p*<0.001), but the loss of body weight was not significant from that of the mono-DDP therapy.

### Autophagy was induced by DDP and suppressed by CQ in the hypopharyngeal xenograft tumors

**Fig 2:**
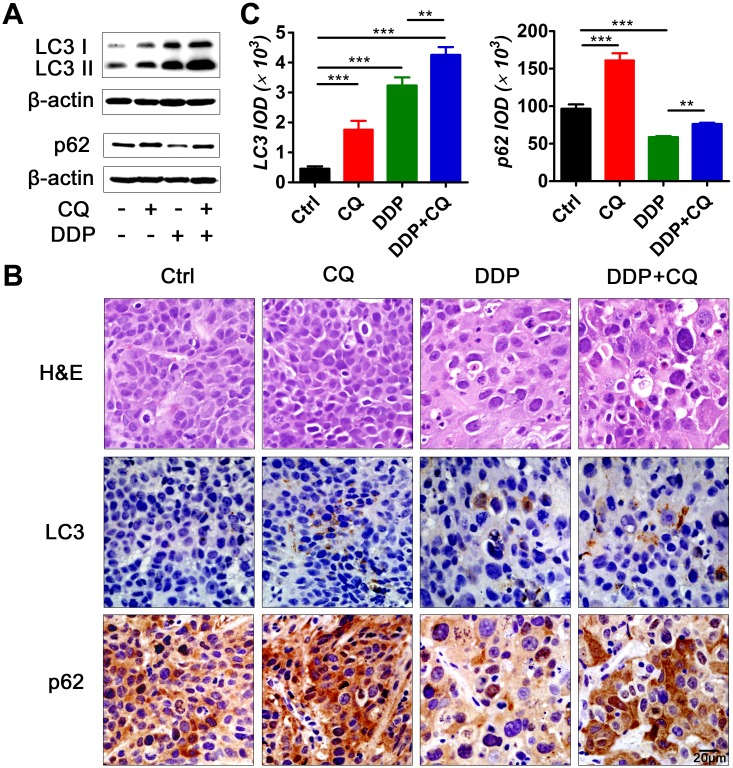
Inhibition of autophagy by CQ and induction of autophagy by DDP *in vivo*. Tumor tissues were harvested for autophagy analysis after treatment for 18 days. (A) Western blot analysis of autophagy markers LC3 and p62. (B) Representative micrographs of tumor sections: hematoxylin and eosin (H&E) stain (upper panel) and immunohistochemistry (IHC) for LC3 (middle panel) and p62 (lower panel). Scale bar = 20 μm. (C) Quantification of LC3 expression (left) and p62 expression (right) according to the Integrated Optical Density (IOD) (**p<0.01, ***p<0.001).

We used LC3 and p62 as markers of autophagy induction and inhibition. Groups of mice were sacrificed and tumor tissues were collected after 18 days of treatment, and the expression levels of LC3 and p62 were examined by Western blot and IHC (Fig 2, S2 Fig). (1) In the mono-CQ treated group, the levels of LC3 and p62 were substantially increased compared with that of control, suggesting a blockade of the autophagy flux by CQ administration. (2) In the mono-DDP treated group, the levels of LC3 were increased whereas accumulation of p62 was decreased relative to control, which suggested that DDP, as a classical anti-tumor agent, caused autophagy induction in the tumors. (3) In the CQ+DDP treated group, a further increase of LC3 levels was observed compared with the mono-DDP treated group.

### CQ addition increased apoptosis in DDP-treated xenograft mice

**Fig 3:**
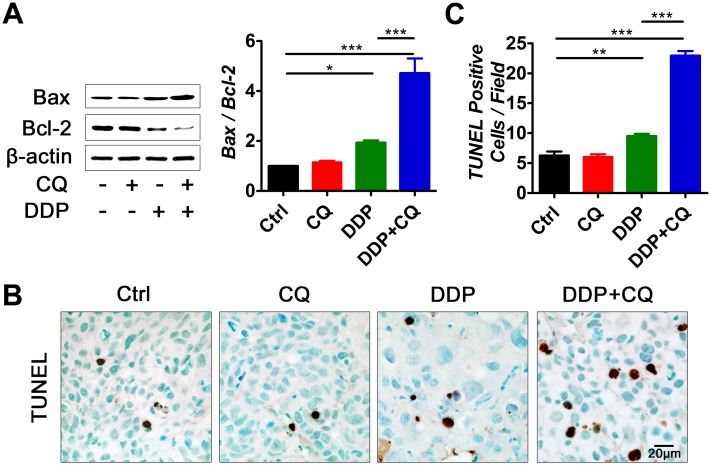
Addition of CQ to DDP treatment increases apoptosis of the tumor cells *in vivo*. (A) Representative photographs of Western blot analysis of Bax and Bcl-2 and Bax/Bcl-2 ratio levels by densitometric analysis of the Western blot bands (7 animals per group). (B) Representative TdT-mediated dUTP nick end labeling (TUNEL) staining of tumor tissues, scale bar = 20 μm. (C) The average numbers of TUNEL-positive cells per field in each group. Cells were counted in 10 randomly selected fields in 7 tumor samples from each group. The values represent the mean ± SEM. *p<0.05, **p<0.01, ***p<0.001.

Bcl-2 family proteins are the main regulators of the mitochondrial pathway of apoptosis. Bax is pro-apoptotic and Bcl-2 is anti-apoptotic [29]. To assess the effect of CQ on tumor cell apoptosis, we performed Western blot analysis of Bax and Bcl-2, and the Bax/Bcl-2 ratio was calculated. A TUNEL assay was also performed to evaluate apoptosis (Fig 3). As expected, DDP treatment caused marked apoptosis of tumor cells (*p*<0.05 relative to the control). CQ alone did not have a pro-apoptosis effect. However, the addition of CQ significantly increased the apoptosis of tumor cells induced by DDP compared with that of DDP alone (*p*<0.001).

### Suppression of Beclin-1 expression by shRNA enhanced the effect of DDP on xenograft tumor growth

**Fig 4:**
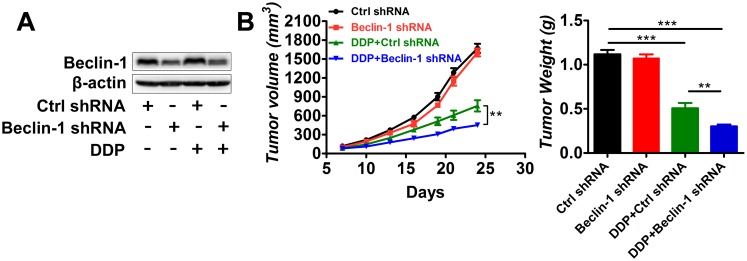
Suppression of Beclin-1 by shRNA decreases tumor growth *in vivo*. FaDu cells infected with lentiviral shRNA to Beclin-1or scrambled control were used for animal model building (n = 7 for each group). Established tumors were treated with DDP (5 mg/kg/6 days) or vehicle control. (A) Western blot of Beclin-1 suppression by shRNA. (B) Tumor volumes and tumor weights for each group. **p<0.01, ***p<0.001.

To further confirm the importance of autophagy inhibition in sensitization to DDP treatment, we inhibited autophagy in FaDu cells with shRNA to Beclin-1 and assessed the effect on xenograft tumor growth. Beclin-1 expression was blocked by shRNA interference in xenograft tumors (Fig 4A). Similar to the results of CQ treatment, suppression of Beclin-1 expression by shRNA did not impact tumor growth (Fig 4B). However, the combination of Beclin-1 suppression and DDP treatment significantly decreased tumor growth compared with DDP plus scrambled control shRNA, as evidenced by the tumor volume and tumor weight data (*p*<0.01).

### CQ delayed autophagy inhibition effects in xenograft tumors

**Fig 5:**
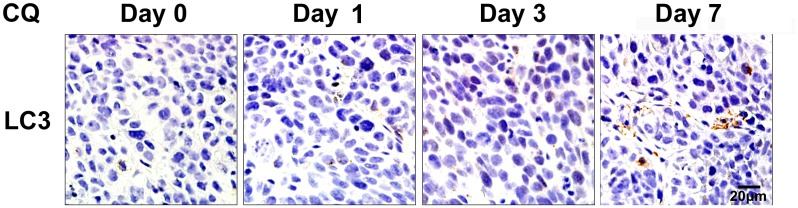
Delayed effects of autophagy inhibition by CQ in xenograft tumors. Tumors treated with CQ (60 mg/kg/day) were collected after treatment for 1, 3 and 7 days and sectioned for LC3 immunohistochemistry. Scale bar = 20 μm.

To confirm the time needed for inhibition of autophagy, tumor bearing mice treated with CQ (60 mg/kg/day) were sacrificed after consecutive administrations for 1, 3 and 7 days separately. Tumor tissues were sectioned for LC3 Immunohistochemistry (Fig 5). LC3 accumulation on day 1 and 3 was rare. A marked accumulation of LC3 was observed after CQ administration for 7 consecutive days, suggesting an autophagy inhibition effect by CQ till day 7.

## Discussion

Previously, we reported that levels of Beclin-1 and LC3 were downregulated in the human HSCC tissues and low expression of beclin-1 and LC3 could correlate with poor prognosis for patients [28]. Both Beclin-1 and LC3 are closely related to the process of autophagy. In this study, we demonstrated that CQ, a mostly used autophagy inhibitor, could inhibit autophagy and increase apoptosis in DDP-treated hypopharyngeal tumor-bearing mice. CQ enhanced the efficacy of DDP, leading to decreased tumor growth and prolonged survival of mice. The inhibition of autophagy by lentiviral shRNA to Beclin-1 had an effect similar to that of CQ administration. Autophagy is a complex cellular homeostatic process. The role of autophagy in cancer is still controversial, and it seems to have both antitumorigenesis and protumorigenesis effects. Though we are still in the early stages of understanding autophagy, targeting autophagy is becoming a new approach for treating cancers. The addition of CQ has been reported to enhance the effectiveness of antitumor therapies in several tumors, including hepatocellular carcinoma [10], colorectal carcinoma [13], esophageal carcinoma [15], prostate carcinoma [8] and breast carcinoma [14]. However, CQ does not sensitize 4T1 tumors [17] or small cell lung cancer [18].

Many *in vitro* antitumor studies have shown evidence of autophagy inhibition by CQ and have mainly attributed the enhancement of antitumor efficacy by CQ to the suppression of autophagy [30]. However, few antitumor studies have provided evidence of autophagy inhibition by CQ *in vivo*. It is often unclear whether CQ reaches sufficient levels in the tumor tissues to effectively inhibit autophagy. Our data demonstrated that CQ administration (60 mg/kg/day) caused significant autophagy inhibition in hypopharyngeal tumor-bearing mice after 18 days of treatment, as indicated by the increased accumulation of LC3 and p62 proteins (Fig 2). However, CQ failed to generate an autophagy inhibition effect at the start of the treatment. The LC3 level of days 1 and 3 did not differ from those of the control on IHC sections (Fig 5), which is in agreement with pharmacokinetic studies on CQ. The maximal plasma concentration of CQ (daily administration) falls within the range of 1.5 and 3 μM. Based on the *in vitro* CQ concentrations required for autophagy inhibition, that concentration is unlikely to effectively inhibit autophagy *in vivo* at the beginning of the treatment [17]. However, CQ has a long half-life and can accumulate to higher concentrations in various tissues over time, especially when it is administered for a long period of time. In our study, it required approximately 7 days for CQ (60 mg/kg/d) to accumulate sufficient concentrations to cause obvious LC3 accumulation in hypopharyngeal tumors. Therefore, it might be necessary to develop a more potent but less toxic autophagy inhibitor that can suppress autophagy *in vivo* at the beginning of intervention.

Although it effectively suppressed autophagy in hypopharyngeal tumors, CQ alone did not affect tumor growth compared with that of control. This result was in agreement with studies on non-small cell lung cancer [31]. In contrast, CQ administered as a mono-drug therapy was reported to cause obvious tumor suppression in pancreatic cancers both *in vitro* and *in vivo* [16]. This finding can be explained by the following. Pancreatic cancer cells have a relatively high level of basal autophagy and, even under normal conditions, rely substantially on autophagy for survival [16,32]. As in HSCC, the levels of Beclin-1 and LC3-II are often downregulated [28], suggesting a downregulation of basal autophagy activity. In other words, hypopharyngeal cancer cells do not have to rely heavily on autophagy to survive under normal conditions. Therefore, when CQ was added alone, autophagy was inhibited, but hypopharyngeal cancer cells could survive and there was no tumor suppression effect.

We also showed that autophagy was induced in tumors treated with DDP. The literature has demonstrated that chemotherapy agents can stress tumor cells and induce autophagy [33]. Tumor cells under stressful conditions may rely on autophagy for survival and to generate resistance to antitumor therapies [34,35]. When treated with DDP, hypopharyngeal cancer cells were severely stressed and had to resort to autophagy for survival. When CQ was added to the DDP treatment, autophagy was blocked and hypopharyngeal tumor cells were unable to utilize autophagy to promote survival. Tumors in the DDP+ CQ group had increased apoptosis compared with the DDP alone group (Fig 3). Tumor growth was decreased and the survival of mice was prolonged by the addition of CQ to DDP (Fig 1). Similar results were observed in the tumor-bearing mice with Beclin-1 suppression (Fig 4), suggesting that autophagy inhibition may enhance hypopharyngeal tumor sensitivity to DDP.

However, it is inappropriate to conclude that the increase in apoptosis and enhancement of efficacy with the addition of CQ only resulted from an inhibition of autophagy. In fact, CQ may even enhance the efficacy of chemotherapy independent of autophagy [36]. As an old drug, CQ still has alternative mechanisms for facilitating cancer therapies. For example, CQ could form a complex with DNA and cause defects in DNA synthesis and repair [37]. It could decrease the sequestration of anti-cancer drugs in endosomes by increasing the endosomal pH, thereby increasing the cytotoxic effects on tumor cells [38]. CQ could induce tumor cell differentiation and inhibit growth [39]. It could also cause tumor vessel normalization and restrain tumor invasion and metastasis while improving chemotherapy [40]. More studies should be performed to explore the detailed mechanisms of CQ-induced sensitization to chemo- and radiotherapies in various tumors.

It is important to note that the combination of CQ and DDP failed to completely control tumor growth. As shown in Fig 1, the tumors finally grew to a large volume and the mice died. This finding may be because CQ was not able to block autophagy to a more severe and complete degree, which allowed the tumor cells to use the residual autophagy for life and avoid death, or because the combination of CQ with DDP therapy was too weak, indicating that more antitumor drugs should be combined with CQ to further strengthen the antitumor effect.

## Conclusions

We demonstrated that CQ increases the efficacy of DDP in treating hypopharyngeal cancers in xenograft mice, resulting in decreased tumor growth and the prolonged survival of mice. Inhibition of autophagy by CQ caused increased apoptosis in DDP-treated tumors. Our study provides support for clinical trials using CQ as an adjunctive antitumor therapy and may help improve human HSCC treatment. However, more studies should be performed to further elucidate the mechanism of CQ-induced sensitization to antitumor therapies.

## Supporting Information

S1 Fig Flow charts for scheduling of the drugs. CQ (60mg/kg/day) was administered intraperitoneally (i.p.) for 18 consecutive days. DDP (5 mg/kg) was administered every 6 days. 3 injections were given in total.
(TIF)

S2 Fig LC3 II levels by densitometric analysis of the Western blot bands. *p<0.05, ***p<0.001.
(TIF)

S1 Table The Guidelines Checklist for Animal Research: Reporting In Vivo Experiments (ARRIVE). The Balb/c nude mice were used for the in vivo experiments, all the related information was provided.
(PDF)
