# Supplementary figures and images for: Chloroquine-Enhanced Efficacy of Cisplatin in the Treatment of Hypopharyngeal Carcinoma in Xenograft Mice
Source: PLoS One. 2015 Apr 29;10(4):e0126147. doi: 10.1371/journal.pone.0126147 (PMC4414471; doi:10.1371/journal.pone.0126147)

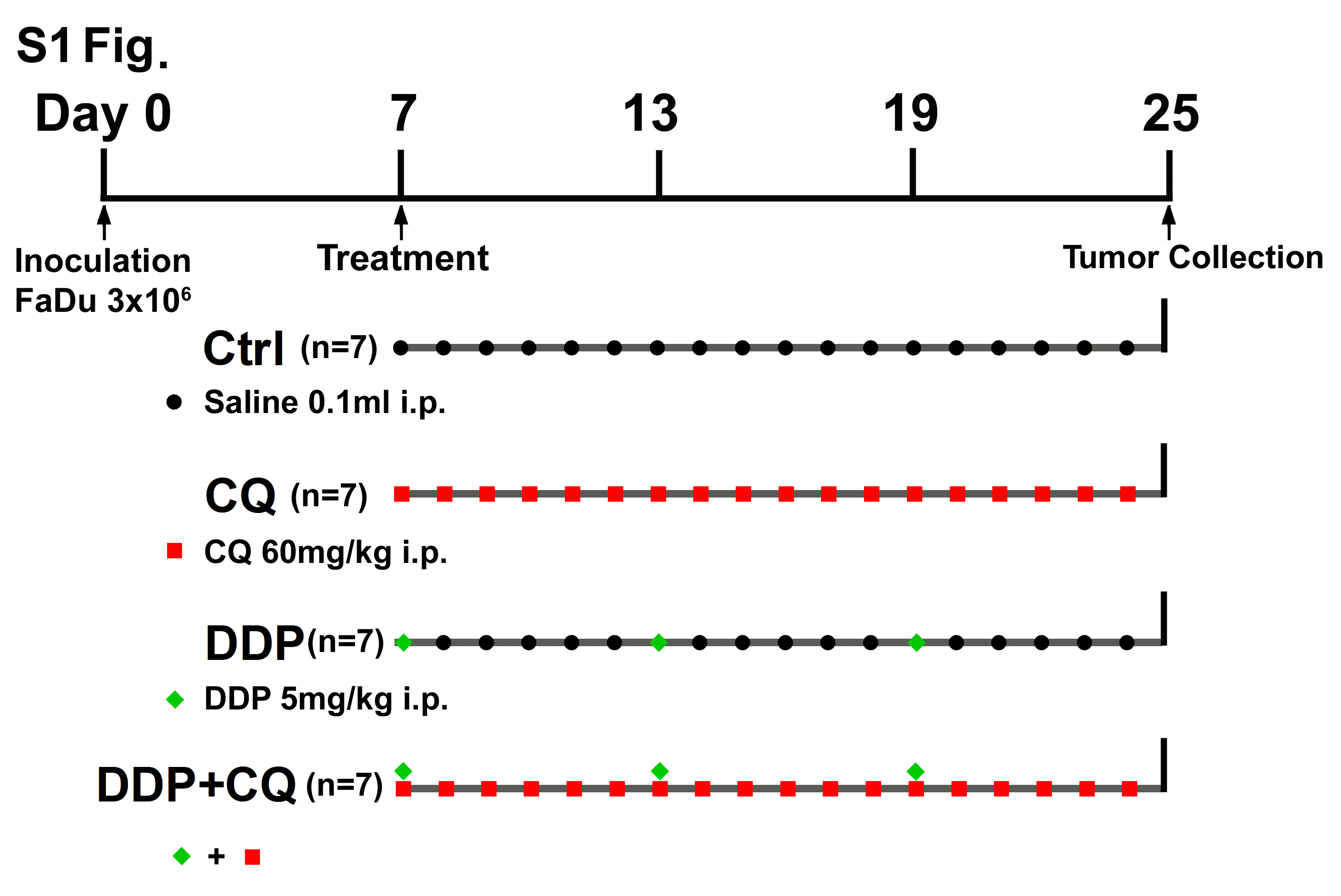

Supplement: S1 Fig — CQ (60mg/kg/day) was administered intraperitoneally (i.p.) for 18 consecutive days. DDP (5 mg/kg) was administered every 6 days. 3 injections were given in total. (TIF) [file pone.0126147.s001.tif]

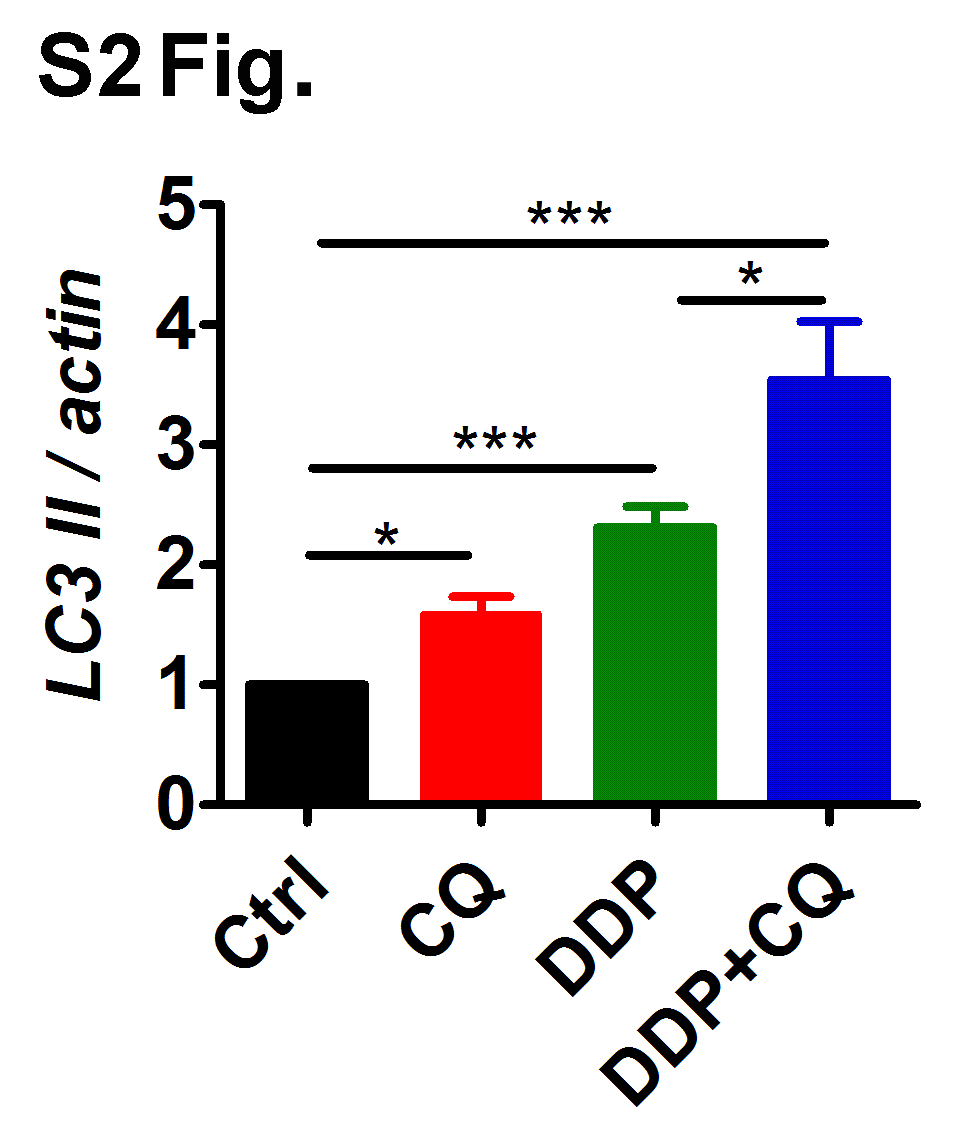

Supplement: S2 Fig — *p<0.05, ***p<0.001. (TIF) [file pone.0126147.s002.tif]
